# Community perception of the autodissemination of pyriproxyfen for controlling malaria vectors in south-eastern Tanzania

**DOI:** 10.1186/s12936-023-04773-2

**Published:** 2023-11-03

**Authors:** Felista S. Tarimo, Angel Dillip, Efraim M. Kosia, Dickson W. Lwetoijera

**Affiliations:** 1 Environmental Health and Ecological Sciences Department, Ifakara Health Institute, P. O. Box 53, Ifakara, United Republic of Tanzania; 2 School of Life Sciences and Bio Engineering, The Nelson Mandela, African Institution of Science and Technology, P. O. Box 4447, Tengeru, Arusha, United Republic of Tanzania; 3 Apotheker Health Access Initiative, P. O. Box 70022, Dar es Salaam, United Republic of Tanzania

**Keywords:** Autodissemination, Pyriproxyfen, Malaria, Community perception, Tanzania

## Abstract

**Background:**

The efficacy of the autodissemination of pyriproxyfen to control malaria vectors has been demonstrated under semi field environment in Tanzania. However, the information on how best communities should be engaged for its routine and large-scale adoption are lacking. This study assessed the community’s level of knowledge, perceptions, acceptability of the autodissemination of pyriproxyfen, and the perceived risks on the safety of pyriproxyfen on the environment.

**Methods:**

This was a concurrent mixed methods study, comprised of a community-based survey of 400 household representatives and eight focus group discussions (FGDs). The study was conducted in two villages in Mlimba district in south-eastern Tanzania between June and August 2022. For the quantitative data analysis, descriptive statistics were applied using R software, while inductive approach was used for qualitative data analysis, using NVivo software.

**Results:**

Knowledge on autodissemination of pyriproxyfen approach was found to be relatively low among both the FGD respondents and surveyed community members (36%, n = 144). Nevertheless, when it was explained to them, the envisioned community support for the autodissemination approach was relatively high (97%, n = 388). One of the major perceived benefits of the autodissemination of pyriproxyfen was the reduction of malaria-transmitting mosquitoes and associated malaria transmission. Environmental impact of pyriproxyfen on non-target organisms and health risk to children were among the major concerns. When provided with information on the safety and its utilization particularly through autodissemination approach, 93.5% (n = 374) of the survey respondents said that they would allow the PPF-contaminated pots to be placed around their homes. Similarly, FGD respondents were receptive towards the autodissemination of pyriproxyfen, but emphasized on the need for raising awareness among community members before related field trials.

**Conclusion:**

This study indicates a low knowledge but high support for scaling up of the autodissemination of pyriproxyfen as a complementary tool for malaria control in rural Tanzania. The Findings of this study suggest that community sensitization activities are required to improve the community’s acceptability and trust of the approach before respective field trials.

## Background

In 2021 alone, malaria caused an estimated 247 million cases worldwide, of which 234 million cases, approximately 96%, were from the World Health Organization (WHO) African Region [1]. Tanzania is one of four sub-Saharan African countries that account for more than half of all malaria deaths worldwide, and it is responsible for 4% of all deaths [1].

Tanzania’s National Malaria Control Programme aims to ensure that all operational health facilities provide accurate diagnostics and treatment with recommended anti-malarial medicines [2]. In addition, the programme facilitates the distribution of long-lasting insecticidal nets (LLINs) and anti-malarial drugs at reproductive and child health clinics [2]. Other malaria control interventions, such as indoor residual spray (IRS) and larval source management (LSM) through the application of bio-larvicides are provided at the community level through different mechanisms [2].

Despite the implementation of multiple strategies, achieving complete control of malaria vectors in endemic settings like Tanzania remains difficult. Among other factors, this is caused by shift in mosquito behaviours, such as changes in host preferences and resting behaviours [3–5]. In addition, there is a continuous development of resistance within target mosquito population to common insecticides like pyrethroids in LLINs [5–7], ensuring their survival and successful transmission of malaria parasites to humans.

As the result, larval source management has been recommended as a complementary intervention, particularly in malaria-endemic areas, to accelerate malaria elimination efforts by 2030 [8]. One of the ways that LSM can be utilized is through larviciding, which involves the regular application of biological or chemical insecticides to water bodies [8]. Insect growth regulators (IGRs), such as pyriproxyfen (PPF), has been proven effective for larviciding [8–10].

Pyriproxyfen (PPF), is a juvenile hormone analogue (JHA) that interferes with the mosquito metamorphosis process, preventing the emergence of adult mosquitoes. Pyriproxyfen presents desirable characteristics of insecticides either for both convectional larviciding and the autodissemination approach [10–12]. In addition to larviciding, recent studies indicate that the combination of pyriproxyfen and pyrethroids in a net can enhance contact mortality, decrease reproductive outputs, and reduce the lifespan of susceptible and pyrethroid-resistant mosquitoes, leading to a decrease in malaria incidence [13–16].

Pyriproxyfen has been approved by the WHO as the larvicide in the control of insects of the public health concern for the past three decades [17, 18], with the recommended limit of 300 ppb in human drinking water, which is 3 to 5 times higher than the amount (50–100 ppb) recommended for mosquito control programme [19]. At extremely low level, pyriproxyfen is highly selective, only targeting specific insect species during their developmental process [17, 20, 21]. While some studies have reported minor negative effects on non-target species, others have found no significant impacts [11, 22, 23]. However, considering the low dosage of PPF that can be naturally transferred by contaminated mosquitoes, it would be unlikely to raise its concentration in breeding habitats to a level that would significantly impact non-targeted organisms, but yet enough to control targeted mosquitoes.

The autodissemination approach relies on adult mosquitoes exposed to contaminated resting sites to disperse the picked insecticide to larval breeding habitats, and disrupt normal mosquito developmental processes [24]. The approach has the potential to contribute towards challenges of convectional larviciding such as difficulties to identify productive breeding habitats and lack of skilled personnel and resources to perform larviciding, and potential to complement conventional larviciding and negatively impact vector populations [25, 26]. The efficacy of autodissemination with PPF has been demonstrated across a range of disease vectors such as *Aedes aegypti* and *Aedes albopictus* [27, 28], *Anopheles gambiae* [29] and *Anopheles arabiensis* [30]. Pyriproxyfen can remain effective under suitable field conditions up to 6 months [20], reducing the need for multiple applications, making the approach suitable for resource limited areas and where malaria is endemic.

The potential of autodissemination of PPF for malaria control in south-eastern Tanzania has been investigated for nearly a decade, and promising findings have been demonstrated [30–32]. In addition, pre-requisite information to guide implementation of the approach under real life settings have been generated, including baseline surveillance of malaria vectors to assess their host-seeking and resting behaviours, as well as investigate their aquatic habitats’ larval productivity. However, innovative ways of engaging the communities for successfully scaling-up of the approach is yet to be explored. Therefore, the present study investigated and documented knowledge, awareness and perceptions of community members on the use of autodissemination with PPF for malaria control.

## Methods

### Study area

**Fig. 1:**
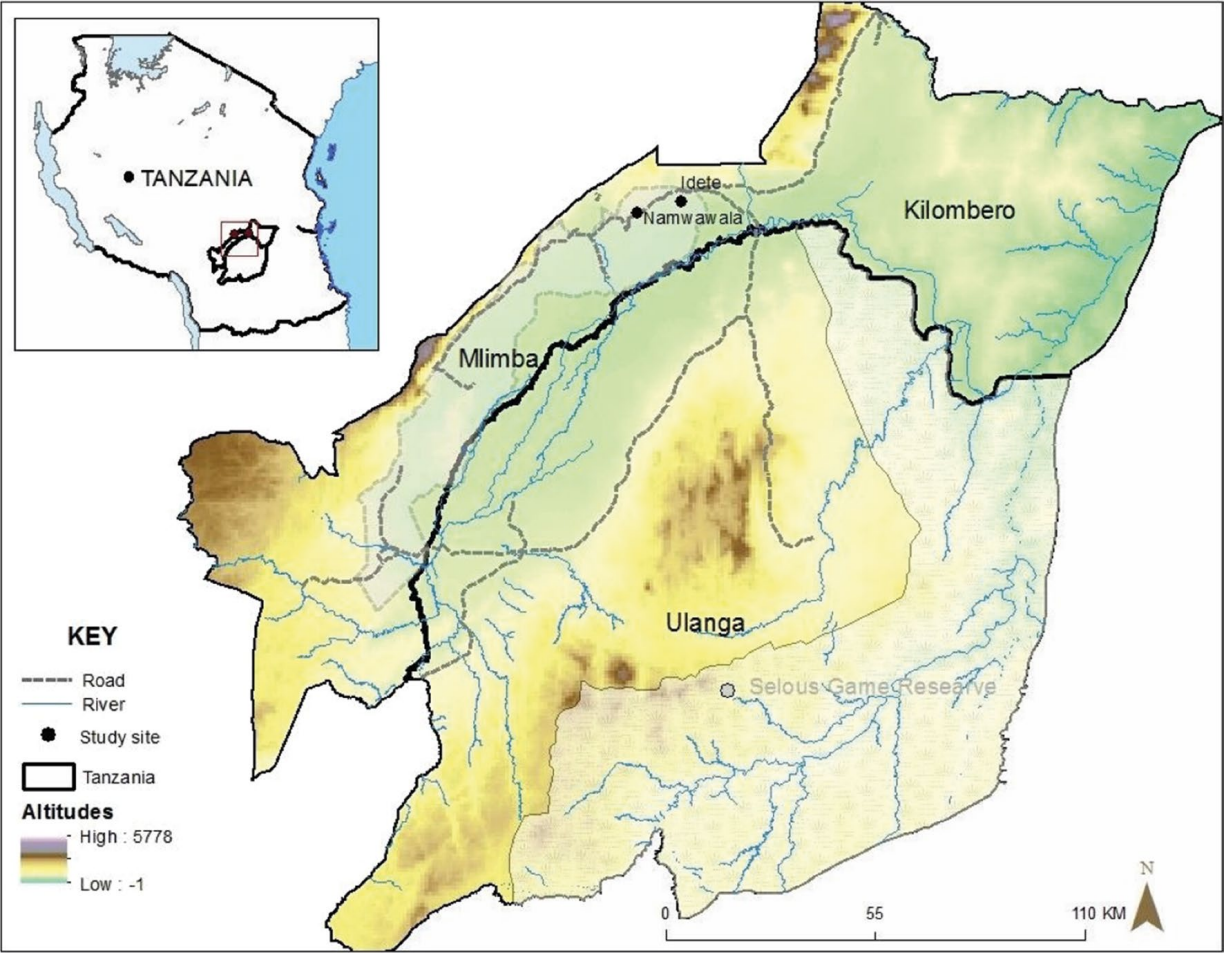
A map showing Idete and Namwawala wards in Mlimba district, south-eastern Tanzania, where the study was conducted

The study was conducted in Namwawala and Idete villages of Mlimba district council in south-eastern Tanzania (Fig. 1). Detailed description of the study villages is provided by Lwetoijera and others [33]. The majority of the communities are subsistence farmers of rice and maize, and they practice cattle farming and fishing at a small scale. The climatic conditions of these villages provide an ideal habitat for mosquito species such as *An. arabiensis* and *Anopheles funestus*, with an Estimated Inoculation Rate (EIR) (bites per person per year) of 9.19 [34]. Studies indicate a preference on both species for human blood meals, but *An. arabiensis* exhibits a more opportunistic behaviour by equally feeding on other animals as well [35]. Their breeding habitats range from large vegetated permanent or semi-permanent water sources that are preferred by *An. funestus* to small vegetated puddles preferred by *An. arabiensis* [36, 37]. Since there are many and diverse breeding habitats in rural areas, locating and treating them can present significant challenges. The efficacy of novel approaches like autodissemination of pyriproxyfen has been shown to be promising in addressing these situations [30, 38].

### Study design and data collection

A concurrent triangulation mixed method approach [39] was used. Data were collected through household surveys and focus group discussions (FGDs) between June and August 2022. The survey questionnaire was used to assess the levels of knowledge, perceptions and acceptance of the autodissemination of pyriproxyfen approach for malaria control among community members. After obtaining information from village leaders regarding the number of households in their respective villages, a total of 400 households were selected from the study population proportionately. The structured questionnaire was administered to an adult member of the selected household, alternating between males and females to ensure gender consideration in community’s perspectives. Data were recorded on electronic tablets using KobotoolboxTM software [40].

FGDs were used to explore in-depth perceptions of community members on the autodissemination of pyriproxyfen approach and its potential for malaria control, and were conducted with farmers, pastoralists, community leaders and primary school students. Purposeful sampling was used to recruit participants. To recruit students who participated in the study, two primary schools; one from each village were selected. In each school, two groups were formed, one of class five and other of class six, and further divided into separate groups of boys and girls to ensure equal representation of views. After informing the students about the objectives of the research, those who expressed interest to participate by raising their hands were selected at random and once assented, they were provided with informed consents to give their parents. Those whose parents agreed by signing the consent forms participated in the discussion.

A total of eight FGDs were carried out until data saturation was observed. Each session included male and female participants, and lasted around 2 h. All discussions were conducted in Swahili language and they were audio recorded.

The survey instrument and focus group discussion guides were piloted in Idete village. Except for children who participated in FGDs, participants’ consent was requested on the day of data collection, after being provided with a detailed description on the purpose of the survey and discussions.

### Data processing and analysis

Quantitative data was analysed using R statistical software 3.3.2 [41]. Descriptive analysis was used to assess socio-demographic characteristics of the survey respondents, and summarize the responses from the community members regarding the autodissemination of pyriproxyfen.

For the qualitative data, audio recordings from the FGDs were transcribed immediately following the discussions. The transcripts were reviewed and analysed using NVIVO 12 Plus software [42]. The study objectives, discussion guides, and a comprehensive review of the transcripts were used to develop inductive codes during the analysis. The analysis was conducted in Swahili, and only selected quotes were translated to English.

Integration was done after both types of data were analysed. Quantitative findings from the survey were presented first, and explanations or direct quotations were given from the FGDs to further describe the themes.

## Results

### Demographic characteristics of study participants

Table 1 shows the demographic details of 400 survey respondents. The mean age of the respondents was 46.61 years. Females accounted for 56.25% (n = 225) of the respondents. The mean number of individuals and children under 5 years of age living in each household was 4.8 and 0.8 respectively. The majority of households 47.9% (n = 192) relied on open wells for their daily water supply, while 26.2% (n = 105) relied on piped water and 25.9% (n = 104) on pumped water. All water sources were categorized based on WHO Joint Monitoring Programme for water supply and sanitation classification [43].

**Table 1:** Demographic characteristics of survey participants

Variables	Category		Frequency (%)
Age (years)			Mean 46.61 (S.D 15.40)
Gender	Males		175 (43.75%)
	Females		225 (56.25%)
Highest level of education	Never attended school		66 (16.50%)
	Primary		291 (72.75%)
	Secondary		40 (10.00%)
	Certificate and higher		3 (0.75%)
Marital status	Single		17 (4.25%)
	Married		332 (83.00%)
	Widowed		37 (9.25%)
	Divorced		14 (3.5%)
Occupation	Farmers		360 (90%)
	Pastoralist and farmer		26 (6.50%)
	Others		14 (3.5%)
Household monthly expenditure	Less than 200,000		82 (20.50%)
	200,000–400,000		293 (73.25%)
	400,000 and above		25 (6.25%)
Toilet type	No/open defecation		1 (0.25%)
	Pit latrine		168 (42.00%)
	Indoor flush toilet		10 (2.50%)
	Outdoor flush toilet		221 (55.25%)

*S.D* standard deviation

For qualitative data, of the 79 participants that were involved, 23 were students (13 boys and 10 girls), and 56 were adults (44 males and 12 females). The average age of adult participants was 46.4 years, ranging from 22 to 75 years, while the average age of students was 12.9 years, ranging from 11 to 18 years. Of the 56 adults, two had a university education, seven had a secondary education, 40 had a primary education and seven had never attended school. Furthermore, 47 of them were married, seven were single, one was divorced and one was a widow.

### General knowledge on mosquitoes and malaria

Of the survey respondents, 97% demonstrated awareness of mosquitoes as disease vectors, with 86.25% (n = 397) specifically identifying malaria among diseases spread by mosquitoes. About 78.00% (n = 312) of respondents were knowledgeable that malaria is transmitted by *Anopheles* mosquitoes. Nearly all respondents 99% (n = 396) agreed that people can be protected against mosquito bites, and the majority 99.7% (n = 395) reported to use LLINs. Other protective measures included repellent oils 14.6% (n = 58), treating of breeding habitats with larvicides 1.5% (n = 6), manipulation of breeding habitats 6.5% (n = 26), burning kits which contains insecticides 4.2% (n = 17) and insecticides 1.5% (n = 6).

Similarly, the majority of FGD participants expressed a good understanding about mosquitoes and malaria transmission. School children had a good understanding of how mosquitos reproduce, grow, and transmit malaria from one person to another, and they reported to acquire such knowledge from school teachings. For example, one student explained that malaria transmission takes place when a mosquito bites an infected person and then pass on the parasites when it next bites a healthy person:

*“A mosquito is an insect that can bite you, if it bites a person with malaria and comes to bite you, it can leave you with malaria parasites.” (Female Student, 13).*

Survey respondents mentioned areas that they thought mosquitoes could breed, and they frequently mentioned puddles 88.7% (n = 299), water filled containers 59.9% (n = 202), septic tanks and toilets 37% (n = 126), brick pits 8% (n = 27) small forests characterized with shrubs 33% (n = 112), and marshes 31% (n = 103). FGD participants identified similar features, with a particular focus on toilets and septic tanks. They indicated that earth brick-making contributes to the creation of breeding habitats in the community, as this participant said:

*“There are those breeding habitats caused by human activities when they make earth bricks, you find when the rain comes, they become filled water that can last for an entire year until when it rains again the next year.” (Female community member, 44).*

### Risk of mosquito bites and malaria transmission

Focus group discussion participants explained that they were at high risk of mosquito bites between sunset and midnight. At home, women were reported to be more at risk of mosquito bites because they spend the early night hours outdoors doing various household chores such as cooking, while men are more likely to be indoor waiting for dinner, watching television, movies, football, or they might be away socializing with other men in the community. On the other hand, men reported to be more vulnerable to mosquito bites when engaging in activities such as farming, fishing, and cattle herding. The participants explained that such activities often expose people to areas with many malaria mosquitoes, and where the use of bed nets is difficult, as this participant explained:

*“There’s that season, maybe you have gone camping; there are mosquitoes there, but you can’t take a mosquito net … because the camping areas are along the rivers, and grassy areas.” (Male community member, 37).*

Only 34% (n = 136) of the survey respondents indicated that mosquitoes biting outdoors can transmit malaria. The FGD participants also shared that not all mosquitoes can transmit malaria. It was unanimously agreed among the majority of participants that mosquito bites that occur in the evening and early night hours do not transmit malaria, and that malaria can be transmitted only by mosquitoes that bite at midnight as this participant elaborated:

*“Malaria mosquito often bites in the middle of the night. And it is a very dangerous mosquito. Under normal circumstances you may be sleeping under the net…but it can also be at 2 AM while going out to the bathroom, when you lift up the net to go out, mosquitoes can get inside the net. When you come back, they attack you.” (Male community member, 50).*

### Challenges with the current malaria interventions

FGD participants acknowledged that bed nets alone are inadequate in controlling malaria due to various challenges, including nondurable net materials that necessitate frequent replacement before recommended lifespan of LLINs. Additionally, respondents stated that the available LLINs do not fully protect them, because individuals still experience mosquito bites while inside the net. It was also reported that mosquito nets are expensive in the retail market. While the majority relies on free-distributed nets by government, they emphasized that those nets are not sufficient, and they proposed its distribution to consider the size of family. One of the community members said:

*“These long-lasting insecticide-treated nets have become expensive for us who buy in stores. It is good that there are those distributed by the government in hospitals and in schools. But I have also noticed that when the children return home, they don’t use them; instead, the parents use them while the child is left open.” (Male community member, 61).*

Participants recommended supplementing bed nets with other interventions for optimal malaria control. Larval source management was among of interventions discussed, where participants reported to have been clearing long grasses, bushes and water pools near and/or around human dwellings to prevent mosquito breeding and resting. Respondents also suggested enhancing toilet facilities, as the available ones have a poor drainage system, and are in poorly constructed structures which result in unrestricted movement of mosquitoes.

The majority of FGD participants were unaware of any LSM activities in the community, despite the ongoing implementation by the government in these communities. There were community leaders who reported seeing application of larvicides, but they had not been involved as this leader said:

*“I have witnessed that thing…it was right here in Idete. Larvicide was brought and given to a community health worker who went to spray on the ponds that does not usually dry.” (Male community leader, 47).*

### Perceptions about the trend of Malaria

85% (n = 339) of households reported at least one malaria case within the past 12 months. Of these, 84.38% (n = 297) said that they were healed after receiving treatment at a nearby dispensary, while 13.07% (n = 46) went to a nearby laboratory for diagnosis and afterward bought drugs from a pharmacy, and 2% (n = 7) said they went to referral hospital for severe malaria. Only 0.57% (n = 2) reported receiving malaria treatment from either a traditional healer or homemade remedies whenever malaria symptoms were observed. Common malaria symptoms mentioned were headache and body aches, chills, fever, loss of appetite, nausea, vomiting, fatigue, diarrhea, and sometimes convulsion.

48% of the respondents (n = 192) reported noticing a decline in mosquito abundance and malaria cases over past few years, while 30.75% (n = 124) reported an increase in malaria cases and 20.25% (n = 80) reported to have seen no significant difference, while only 1% (n = 4) stated that they did not know. Similarly, FGD participants reported a decrease in mosquitoes and malaria cases, particularly in areas with ongoing research programmes. Those who reported a decrease in mosquito densities attributed the success to improved awareness about mosquitoes and diseases they transmit, importance of environmental management, use of insecticides, wide coverage of LLINs and availability of medicines as this participant said:

“*To be honest, I have noticed a decrease in malaria from when I moved here. It has decreased due to the availability of medicines in hospitals, as well as research and these long-lasting insecticide-treated nets, they have helped a lot.” (Male community leader, 61).*

However, the participants noted that mosquitoes were still a major problem in terms of causing discomfort and spreading diseases as this participant said:

*“Malaria is present! When you don’t have it, someone else has it. The only thing that can help with it is by sleeping in the mosquito net, but people are still having challenges with malaria. As far as I know, malaria exists and mosquitoes exist.” (Male community member, 50).*

Other respondents linked livestock-keeping and increased exposure to mosquito bites. Although they could not provide specific details on the species of mosquito, they reported instances of mosquitoes biting cattle, potentially increasing their capacity to reproduce and transmit malaria, as this participant said:

*“I have noticed that mosquitoes are numerous for us who live with livestock, compared to other communities. Mosquitoes follow the smell of the livestock during the night, they attack cows, you find them full with blood in the morning … they reproduce and become more numerous.” (Male community member, 45).*

Among the listed activities that elevate the risk of exposure to malaria are communal events such as burial ceremonies, weddings and other celebrations as stated by one of the respondents:

*“We often get attacked by mosquitoes during social events, such as in burial ceremonies. We usually don’t use a mosquito net or anything, we just sleep in an open area.” (Male community leader, 40).*

### Knowledge about autodissemination of pyriproxyfen for controlling Malaria vectors

36% (n = 144) of survey respondents provided a correct explanation for the autodissemination of pyriproxyfen, while two thirds 64% (n = 256) were unfamiliar with its meaning. Those who could explain, reported to have learnt about the approach through friends and neighbours, researchers, and community meetings. Despite of the ongoing study in selected communities, few FGD respondents were knowledgeable of autodissemination approach for controlling malaria transmitting mosquitoes. One of the community leaders who attended one of the community meetings said:

*“I believe we have heard about it; Ifakara Health Institute called a meeting and explained that there will be a procedure for putting the insecticide in the pots. They said in the future, and I believe this is now the future.” (Female community leader, 50).*

However, some were not aware, as one of the community leaders said:

*“We only heard yesterday when we were walking with these experts to educate the community; that there is an insecticide that if a mosquito picks it from the pots, shall deliver it to their breeding habitats.” (Male community leader, 52).*

Of importance, autodissemination approach was more known among school students who participated in FGDs, but for them too, they had only known of this through reading consent forms sent to their parents for this particular study. One student was able to correctly explain the autodissemination approach:

*“If you contaminate where a mosquito wants to rest after it has sucked human blood to mature its eggs, she will carry the insecticide, and when she goes to lay in their usual breeding habitats, all its babies will die.” (Male student, 12).*

### Perceptions on the use of autodissemination of pyriproxyfen for controlling malaria vectors

After a description of the autodissemination of pyriproxyfen, nearly all FGD participants indicated their support for its scale up for malaria control. The majority of the survey respondents 93.5% (n = 374), agreed for the PPF- containing pots to be placed in their properties (Table 2). Some of the positive attributes of the autodissemination approach listed included its perceived effectiveness in reducing mosquitoes and malaria, as this participant said:

**Table 2:** Survey responses on perceptions about autodissemination of PPF for controlling malaria vectors

Question	Agree (%)	Neutral (%)	Disagree (%)
Autodissemination should be used to control malaria vectors	94.50	3.50	2.00
Trust that the approach will reduce the abundance of mosquitoes in the community	97.00	2. 25	0.75
Would give permission to researchers to put infected clay pots at their household	93.50	2.00	4.50

*“Because it kills mosquitoes, malaria will disappear. Therefore, the project is good and profitable and we support it 100%.” (Male community leader, 29).*

However, some participants were skeptical about this approach, saying that they do not know if it would be a good approach for the community, and that they would wait and see how it works before they formed their opinions as this participant said:

*“I think that approach is good only if it succeeds. Because right now we are just guessing, it hasn’t arrived yet, but if we succeed in implementing it, it will be good.” (Male community member, 27).*

At the same time, participants were interested in additional details about the potency and duration of pyriproxyfen once applied and wondered whether heavy rains would wash it away before it could take effect. Respondents also indicated that the physical characteristics of the clay pots make them favorable in attracting mosquitoes, hence enough amount of pyriproxyfen would be delivered to the potential breeding habitats. One of the participants said:

*“Mosquitoes prefer cool places, if you use any other thing apart from clay pots during the dry season, they become hot.” (Male community member, 33).*

Even though participants supported the usefulness of clay pots in this project, they advised that it would be important to provide adequate education to community members, as this would prevent theft and misuse once were distributed, as one of the respondents said:

*“Some people may be confused by that pot. Others can wash and start cooking with it without you being aware. My advice is that we educate people about the pot being contaminated with the insecticide and that it is there to control mosquitoes, so no one should take it.” (Female student, 11).*

### Concerns on the safety of pyriproxyfen on the environment

85.5% (n = 342) of survey respondents indicated that larvicides that are used in malaria control programmes are safe, while 13.5% (n = 54) were neutral, and only 1% (n = 4) indicated that they were harmful. Of the four participants in the harmful category, only one of them said larvicides have bad smell, and he reported to have acquired such information through community meetings. The other three said larvicides could cause discomfort and could affect non-targeted organisms sharing breeding habitats with mosquitoes. 95.5% (n = 382) of respondents said they had never heard about pyriproxyfen, while 4% (n = 16) said they had, and 0.5% (n = 2) were not sure. After being provided with a description of the utilization of pyriproxyfen in this research, 72.5% (n = 290), of survey participants expressed that they considered it safe, while 25.5% (n = 102) remained neutral, and only 2% (n = 8) believed it could potentially harm the environment. Out of the eight respondents, four of them expressed concerns regarding its impact on non-targeted organisms, while the remaining were concerned about its potential effects on children.

Similarly, FGD respondents provided supportive viewpoints on the safety of pyriproxyfen. Some respondents within the pastoralists group reported to have participated in previous larviciding activities using pyriproxyfen, and they were informed that pyriproxyfen would neither affect their livestock nor human being. One of the participants said:

*“This is not the first time to use this insecticide. It had already been used, it was sprayed in the pond and it was still being used by the livestock. If no side effects could be found, I believe even in the future they will not happen.” (Male community member, 38)*.

In one of the FGDs, respondents stated the hearsay that the LLINs that were distributed had harmful effects on man’s sexual arousal. They were worried that pyriproxyfen would cause the same, and warned that scientists should not deliver items that will affect the community, as this respondent said:

*“Our concerns began when bed nets were distributed. We have heard that nets deplete male sexual energy! Let us not create things that will be harmful to us in the future. We therefore ask that you pay attention to that.” (Male community member, 33).*

Some of the respondents also expressed concerns and posed questions about the potential impact of pyriproxyfen (PPF) on children in households where contaminated clay pots would be placed. These respondents expressed worries that children might be unintentionally exposed to pyriproxyfen, which could have adverse health effects on them, as this participant said:

*“There will be challenges when the pots are distributed to us. What will happen since we have children? Where should we put the pots so that the children don’t play with them? The children can play with them without knowing the insecticide is toxic, and it may even affect other people in the community.” (Male community member 33).*

Furthermore, students’ perceived risks on the safety of pyriproxyfen ranged from potential personal harm through skin contact while bathing in treated ponds, to livestock and other community members. However, there were respondents who were aware about the fact that the larvicide exclusively targets mosquitoes and does not harm non-target organisms. They were also aware that these larvicides undergo regular safety evaluations in specialized laboratories before being deployed in the field, as one of the students said:

*“The larvicide may not be harmful to living organisms because it is made to kill mosquitoes only, and tests had already been done in the laboratory.” (Male student, 11).*

Upon being provided with an explanation of the safety measures associated with pyriproxyfen and how it is utilized in the ongoing research, they expressed their willingness to support the project by distributing the clay pots throughout the village.

In general, participants stressed on the need to inform and educate the community about the benefits and safety of this approach in order to obtain the community support. Participants suggested that researchers and community leaders to collaborate closely so that information about the project can be easily disseminated to the rest of the community members.

## Discussion

This study demonstrates community’s understanding of mosquitoes, their breeding habitats, malaria transmission, and preventive measures. The majority of individuals recognized the correlation between mosquito breeding habitats and mosquito abundance. Community members agreed that in addition to the current interventions, controlling mosquitoes right at their aquatic stages would contribute to overall reduction of mosquito population. However, there was a lack of knowledge about larviciding, despite its proven effectiveness in controlling malaria [44–46], and being implemented in the district. The knowledge gap among community members might be addressed through designing and improving the community involvement approaches in malaria control programmes.

Although the autodissemination of pyriproxyfen is a new concept in the study area, respondents had an average understanding about its application for malaria control. High level of acceptance and perceived trust in its expected effectiveness by the majority of the respondents was encouraging and of importance for continuing to establish its utility. The fact that respondents learned about the approach through community engagement sessions and researchers working in the communities emphasize the significance of educational efforts in promoting new strategies for malaria control. The findings of this study also suggest that continued research and implementation of the autodissemination approach is a promising addition to malaria control efforts.

Prior to introducing the autodissemination of pyriproxyfen to control malaria mosquitoes, it is crucial to further inform, educate, and engage the beneficiary communities about this intervention. Community members and leaders that participated in the current study have already shown a high level of acceptance for the autodissemination approach. Conducting sensitization sessions is essential to address questions and concerns that raised during discussions about the environmental safety of pyriproxyfen to wide audience. It is particularly important because the targeted communities rely on water sources that have the potential to be contaminated with pyriproxyfen. The engagement of communities plays a key role in ensuring the scaling up and sustainability of novel vector control interventions [47, 48]. These sensitization sessions will not only raise awareness but also ensure that communities make informed decisions, strengthen partnerships and ultimately lead to a greater acceptance of the intervention. Additionally, this process can provide community members with the opportunity to co-design the approach in collaboration with the programme, fostering a sense of ownership and tailoring it to their specific needs and preferences.

While almost all study participants reported using LLINs to protect themselves from mosquito bites, FGD participants argued that the bed nets available within their families are insufficient, resulting in some individuals being left unprotected. While there has been universal distribution of LLINs in the past to cover everyone [49], the ongoing LLINs catch-up and keep-up distributions to pregnant women, children under 5 years and school going children aim to extend protection to these vulnerable populations [49, 50]. With rapid population growth [51], the absence of considerations for family size in distributing LLINs poses a risk of leaving certain community members, particularly those from economically disadvantaged households, without adequate protection [50].

Communities are encountering an additional obstacle in the use of LLINs, which is the diminished ability to effectively control malaria transmitting mosquitoes. This is attributed to changes in the behaviour of the mosquitoes [3, 4, 52], and the development of resistance to insecticides [5–7]. In addition, the nondurable LLINs can easily get torn and enable mosquitoes to penetrate and gain access to the sleeping individuals [53], allowing transmission to continue even in areas with high LLIN coverage. These views are well echoed by most recent suggestions of having mosquito nets that last longer, as that will ensure extended protection against mosquito bites and interrupt malaria transmission [54, 55]. However, it remains important to use LLINs in conjunction with other measures such as LSM and housing improvement so as to reduce the risk of exposure to mosquito bites when bed nets are not used [56, 57], or when its use is compromised by shared small sleeping space or the ability for mosquito to penetrate as results of its small body size [58].

Similar to the previous findings in the same region, participants expressed the risk of outdoor exposure to mosquito bites during various important household activities [52, 59]. Considering the significance of community knowledge in effective control strategies for malaria [47, 48], it is crucial to recognize the threat posed by mosquito bites during the early evenings and early mornings, especially in rural areas [60–63]. This study underlines the importance of integrating community knowledge and understanding of outdoor malaria transmission dynamics into malaria control interventions. By actively involving community members, interventions can be tailored to address their specific needs, beliefs, and practices, thereby increasing utilization and acceptance.

Members of the community indicated that malaria mosquitoes breed in poorly designed toilets and septic tanks, and they strongly advocated for better toilets to control them. While improved toilets are important for preventing waterborne diseases like cholera [64], it is often non-malaria transmitting mosquitoes, such as *Culex quinquefasciatus*, that prefer breeding and resting in toilets [65, 66]. The misconception that malaria-transmitting mosquitoes breed in toilets clearly highlight continued community’s engagement coupled with education on malariology. Furthermore, it is crucial to implement carefully designed and evaluated social and behavioural change communication strategies that specifically target diverse segments of the population, such as the elderly, men, women, and individuals with low literacy levels.

The community members demonstrated awareness that proximity of livestock to human populations could have a substantial impact on malaria transmission [67]. However, the knowledge gap on the differences in feeding patterns among malaria vectors emphasizes the importance of education and community awareness that will be useful in understanding, accepting and effectively use interventions targeting different vector species. Similar views were found in a study conducted in Vietnam, where respondents indicated that keeping livestock near their homes increased the spread of mosquito-borne diseases [68]. However, the impact of livestock keeping in malaria transmission is still debatable [69]. On one hand, livestock may divert the host-seeking mosquito that also prefers to feed on alternative non-human host from human dwellings, thereby decreasing the risk of mosquito bites [70]. Alternatively, if livestock are kept nearby, might draw human-seeking mosquitoes to their vicinity and increase the risk of mosquito bites [71]. All these behaviours can be exploited to target malaria mosquitoes either though mass trapping of malaria vectors that prefer feeding on both human and animals [72–74], direct treatment of cattle and their shelter with topical insecticides such as deltamethrin [72, 75, 76], or endectocides such as ivermectin that kills landing or feeding mosquitoes [77, 78].

The following limitations were encountered during the course of this study. First, acknowledging the limited number of coders consisting of only two individuals, there is a potential of bias in the presentation of the findings. Nevertheless, the themes included in the manuscript reflect the consensus reached by all authors after the coding harmonization session. It is also acknowledged that there is an inherent potential for respondents in qualitative research to provide socially acceptable or desirable responses rather than revealing their true attitudes and behaviours. However, several strategies were implemented to enhance the validity of the data. First, multiple data sources including household survey and FGDs were used to provide a comprehensive understanding on community’s perceptions on the autodissemination of pyriproxyfen. Additionally, pilot testing was conducted to refine data collection tools and identify and rectify potential sources of bias. Reflexivity was also maintained throughout the research process, and rigorous inductive coding was employed during data analysis to ensure that findings were derived from the data itself rather than from preconceived notions.

## Conclusion

This study documented the receptiveness of the communities to autodissemination of pyriproxyfen approach for malaria control in rural Tanzania. The majority of community members who participated in the study expressed their perceived trust in the effectiveness of this approach. They also emphasized the need for community sensitization activities to enhance acceptance and trust among community members before the implementation of field trials for the autodissemination of pyriproxyfen. These activities can serve as a valuable tool to educate and inform the community about the intervention’s benefits and possible risks. By doing so, the community can make informed decisions and feel more involved in the process, which can lead to greater participation and overall success of the intervention.

## Data Availability

The datasets used and/or analysed during the current study are available from the corresponding authors on reasonable request.

## Abbreviations

EIR: Entomological Inoculation Rate
FGDs: Focus group discussions
IHI: Ifakara Health Institute
IRB: Institution Review Board
IRS: Indoor residual sprays
JHA: Juvenile hormone analogue
LLINs: Long-lasting insecticidal bed nets
LSM: Larval Source Management
NIMR: National Institute for Medical Research
PPF: Pyriproxyfen
